# The HIV RNA setpoint theory revisited

**DOI:** 10.1186/1742-4690-4-65

**Published:** 2007-09-21

**Authors:** Ronald B Geskus, Maria Prins, Jean-Baptiste Hubert, Frank Miedema, Ben Berkhout, Christine Rouzioux, Jean-Francois Delfraissy, Laurence Meyer

**Affiliations:** 1 Department of Clinical Epidemiology, Biostatistics and Bioinformatics, Academic Medical Center, Meibergdreef 15, 1105 AZ, Amsterdam, The Netherlands; 2 Department of Internal Medicine, Academic Medical Center, Meibergdreef 15, 1105 AZ, Amsterdam, The Netherlands; 3 Cluster Infectious Diseases, Department of Research, Amsterdam Health Service, Nieuwe Achtergracht 100, 1018 WT, Amsterdam, The Netherlands; 4 Inserm, U822, Le Kremlin-Bicêtre, F-94276, France; 5 AP-HP, Hopital Bicêtre, Epidemiology and Public Health Service, F-94276, France; 6 Department of Immunology, University Medical Center, Utrecht, The Netherlands; 7 Department of Human Retrovirology, Academic Medical Center, Meibergdreef 15, 1105 AZ, Amsterdam, The Netherlands; 8 Department of Virology, Hôpital Necker, Paris, France; 9 Univ Paris-Sud, Faculté de Médecine Paris-Sud, Le Kremlin-Bicêtre, F-94276, France

## Abstract

**Background:**

The evolution of plasma viral load after HIV infection has been described as reaching a setpoint, only to start rising again shortly before AIDS diagnosis. In contrast, CD4 T-cell count is considered to show a stable decrease. However, characteristics of marker evolution over time depend on the scale that is used to visualize trends. In reconsidering the setpoint theory for HIV RNA, we analyzed the evolution of CD4 T-cell count and HIV-1 RNA level from HIV seroconversion to AIDS diagnosis. Follow-up data were used from two cohort studies among homosexual men (N = 400), restricting to the period before highly active antiretroviral therapy became widely available (1984 until 1996). Individual trajectories of both markers were fitted and averaged, both from seroconversion onwards and in the four years preceding AIDS diagnosis, using a bivariate random effects model. Both markers were evaluated on a scale that is directly related to AIDS risk.

**Results:**

Individuals with faster AIDS progression had higher HIV RNA level six months after seroconversion. For CD4 T-cell count, this ordering was less clearly present. However, HIV RNA level and CD4 T-cell count showed qualitatively similar evolution over time after seroconversion, also when stratified by rate of progression to AIDS. In the four years preceding AIDS diagnosis, a non-significant change in HIV RNA increase was seen, whereas a significant biphasic pattern was present for CD4 T-cell decline.

**Conclusion:**

HIV RNA level has more setpoint behaviour than CD4 T-cell count as far as the level shortly after seroconversion is concerned. However, with respect to the, clinically more relevant, marker evolution over time after seroconversion, a setpoint theory holds as much for CD4 T-cell count as for HIV RNA level.

## Background

CD4 T-cell count and HIV RNA level (viral load) are the most widely used markers of progression to AIDS and death in HIV-1 infected persons. Investigating their natural course after infection and the effect of covariates on this natural course is of great importance for prognosis, deciding when to start highly active antiretroviral therapy (HAART), and the understanding of marker dynamics after HAART interruption. It is generally agreed that CD4 T-cell count shows a consistent decline after HIV infection. For the evolution of viral load, the setpoint theory was introduced soon after the implementation of HIV RNA assays [1,2]. Three components describe the setpoint behaviour after the high peak reached in the first few weeks after infection: viral load remains relatively stable for a certain period (the "setpoint"); individuals who have a higher setpoint level have faster AIDS progression; and shortly before the development of AIDS, viral load rises again. A U-shaped curve has been another way to describe the same phenomenon [3]. As a consequence, individuals with faster AIDS progression have a shorter duration of the plateau phase. The definition of the plateau phase has not been consistent and departure from the plateau phase has been defined as an increase of 0.5 to 1 units from baseline level on the base-10 logarithmic scale [1,4,5]. Aspects of the setpoint theory have been criticized [6-10]. Alternative reasons have been suggested for the apparent stable level of viral load, including lack of sensitivity and precision of the assay used [6] or selection of specific subgroups and short follow-up [8].

The predictive value of the level in viral load reached shortly after seroconversion has been shown convincingly before [11,12]. The aim of this study was to reconsider the two other aspects of the HIV RNA setpoint theory by using a method that allows for a direct comparison of the evolution of two or more markers. By evaluating marker evolution on a scale that is related to progression to AIDS, changes in a marker that do not lead to a change in AIDS risk were considered as clinically irrelevant and therefore seen as stable. The evolution of both markers was modeled and compared from seroconversion onwards and also during the last four years prior to AIDS diagnosis.

## Results

### General characteristics

The 400 persons contributed 2192 person-years of follow-up and 166 AIDS events. Average follow-up time was 6.0 years for the Amsterdam cohort (maximum 14.0 years) and 5.4 years for the French cohort (maximum 9.3 years). We had 6761 CD4 and 3807 HIV RNA measurements. Of the latter, 9% (n = 344) were below the detection limit. Only 183 individuals had HIV RNA measurements in the first six months after seroconversion (with a maximum of four per individual). For individuals who started ART during follow-up (n = 202), the average time from seroconversion to ART administration was 4.1 years. Six percent of the records were obtained from individuals receiving ART with at least two drugs (mainly Zidovudine (AZT), Didanosine (ddI) or Zalcitabine (ddC) and 0.5% from patients receiving more than two drugs); 22% were obtained under monotherapy (mainly AZT), and the remaining 72% were obtained from persons while not on ART. ART had no significant effect on CD4 T-cell count, but did affect HIV RNA level (table 1). The estimated effect is slightly lower than the one presented by Hubert et al. [7].

**Table 1:** Estimates of ART effects on base-10 logarithm of HIV RNA level and cube root of CD4 T-cell count

	HIV RNA			CD4		
	effects	95% CI		effects	95% CI	
monotherapy (first 6 months)	-0.32	-0.39	-0.24	0.02	-0.05	0.08
monotherapy (next 6 months)	-0.12	-0.21	-0.02	-0.02	-0.11	0.06
dual therapy (first 6 months)	-0.33	-0.47	-0.18	0.05	-0.06	0.15
dual therapy (next 9 months)	-0.25	-0.42	-0.08	-0.08	-0.22	0.06

### Marker evolution from seroconversion onwards

**Figure 1:**
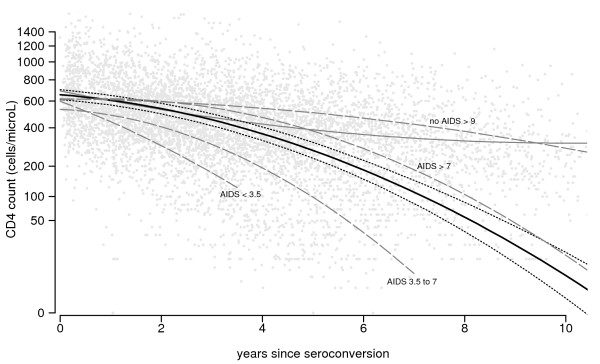
**CD4 count patterns over time after seroconversion**. Scatterplot of CD4 T-cell count values after seroconversion (grey points), together with the fitted least squares curve (i.e. average CD4 T-cell count *values*; thin grey line) and the fitted curve from the random effects model (i.e. average CD4 T-cell count *patterns*; thick black line, with 95% confidence intervals). Average patterns for the groups defined by progression times (in years) are shown as well (dashed grey lines).

**Figure 2:**
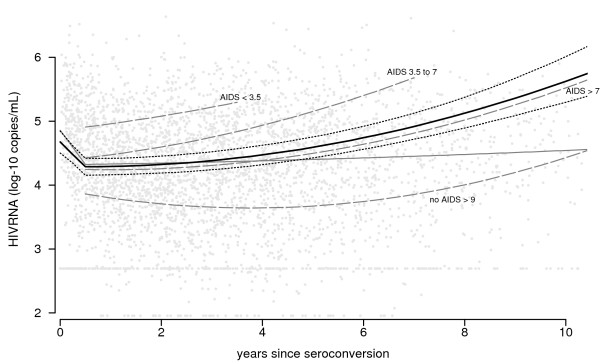
**HIV RNA patterns over time after seroconversion**. Scatterplot of HIV RNA *values *after seroconversion (grey points), together with the fitted least squares curve (i.e. average HIV RNA values; thin grey line) and the fitted curve from the random effects model (i.e. average HIV RNA *patterns*; thick black line, with 95% confidence intervals). Average patterns for the groups defined by progression times (in years) are shown as well (dashed grey lines).

The evolution of CD4 T-cell count and viral load over time after seroconversion is shown graphically in the figures 1 and 2. Since the effects of cube root of CD4 T-cell count and base-10 logarithm of viral load on AIDS risk showed almost no deviations from linearity, this scale is used in the graphs. However, corresponding backtransformed CD4 T-cell counts are shown on the y-axis. Since the effect of changes in CD4 count on AIDS risk is much larger at low counts than at high counts, changes at high counts give smaller changes in the graph.

Individuals with faster AIDS progression have higher HIV RNA level six months after seroconversion. For CD4 T-cell count, this ordering is less clearly present. However, the averaged individual *patterns *for both markers, as derived from the longitudinal approach, are similar after the first six months following seroconversion (thick black lines, with 95% confidence intervals), but of course they trend in opposite directions. Both curves remain fairly stable during the first few years after seroconversion, but curves become increasingly steeper over time. Similarly, for both markers, evolution differs by subgroup as classified by their disease progression: AIDS occurring <3.5 years (N = 40), 3.5 to 7 years (N = 103), >7 years after seroconversion (N = 23), and AIDS-free for more than 9 years after seroconversion (N = 36). Individuals with fast progression to AIDS – i.e., within 3.5 years after seroconversion and, to a lesser extent, between 3.5 and 7 years – do not have a stable plateau phase for either marker. The trends in average *value*, derived from the repeated cross-sectional approach (thin grey lines), are very different from the averaged patterns. Only a modest decrease in CD4 T-cell count is seen, which levels off at a value of around 300 cells/*μ*L after eight years. For viral load, a very small increase in average value is seen (from 4.32 to 4.54). However, the estimated curve at later time points suffers from survivorship bias, since individuals with fast disease progression do not contribute to the estimate.

**Figure 3:**
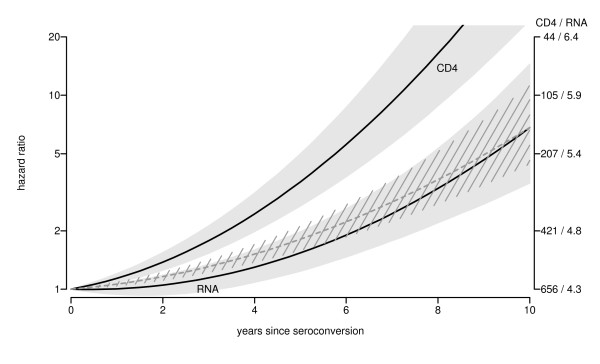
**Marker evolution on the AIDS hazard scale**. Average marker evolution after seroconversion, represented on AIDS risk scale (grey area: 95% CI). Left y-axis shows effects of fitted average marker patterns on AIDS hazard, relative to hazard at average values at seroconversion (656 cells/*μ*L for CD4 T-cell count and 10^4.3 ^= 19952 copies/mL for HIV RNA level). Right y-axis shows corresponding fitted marker values over time after seroconversion. Dashed grey line shows CD4 effect standardized to HIV RNA value ten years after seroconversion (i.e. the CD4 curve is moved downward, such that it corresponds with the HIV RNA curve at ten years after seroconversion; the 95% CI is rescaled as well).

The effects of time-updated marker values on AIDS risk are used to directly compare average patterns of CD4 T-cell count and HIV RNA level on a common scale (figure 3). Numbers on the y-axis are interpreted as follows. Table 2 gives the change in relative AIDS risk per unit change in cube root of CD4 T-cell count and base-10 logarithm in viral load, based on a time-dependent Cox proportional hazards model. Changes in both marker patterns over time after seroconversion correspond to changes in AIDS risk, as shown on the left y-axis. Marker levels at seroconversion (excluding the initial peak for HIV RNA level) have been chosen as reference values (i.e. relative risk 1). Note that a logarithmic scale is used, since this describes the linear effects in a Cox proportional hazards model. For example, over the first four years following seroconversion, the average CD4 T-cell count pattern shows a decline from 656 cells/*μ*L to 364 cells/*μ*L, which implies a drop of 656^1/3 ^– 364^1/3 ^= 1.55 on the cube root scale. This corresponds to a exp(0.5801 × 1.55) = 2.46-fold increase in AIDS risk. The average viral load over the same time span shows an increase by 0.19, corresponding to an exp(1.422 × 0.19) = 1.3-fold increase in risk. In order to double the AIDS risk, the cube root of the CD4 T-cell count should decrease by log(2)/0.5801 = 1.19 (since exp(0.5801 × 1.19) = 2), whereas the base-10 logarithm of viral load should increase by log(2)/1.422 = 0.49. On the right y-axis, the values of CD4 count (on original scale) and viral load (on base-10 logarithmic scale) corresponding with changes in relative risk are shown (again with fitted marker values at seroconversion as reference value). It is seen that the average decrease in CD4 T-cell count over the first ten years induces a much stronger increase in AIDS risk than does the average increase in viral load. Since average viral load remains more stable than CD4 T-cell count on the AIDS-risk scale, one may say that it exhibits more setpoint behavior. However, this difference between the curves actually increases over time after seroconversion: the relative risk ratio RR(CD4)/RR(RNA) increases from exp(0.5801 × 1.55)/exp(1.422 × 0.19) = 1.88 at four years to exp(4.126)/exp(1.919) = 9.09 at ten years after seroconversion.

**Table 2:** Parameter estimates for time-updated marker effects on AIDS risk, based on bivariable model for both markers

	-CD4^1/3^	95% CI	log10 RNA	95% CI
*β*	0.58	0.46–0.70	1.42	1.05–1.79
exp(*β*)	1.79	1.59–2.02	4.15	2.85–5.98

For a direct comparison of the form of both curves, we also show the CD4 trajectory, standardized to the HIV RNA level ten years after seroconversion (i.e. the values in the CD4 curve are divided by 9.09 on the relative risk scale, such that values are the same at ten years after seroconversion). It is seen that, during the first years, average standardized CD4 T-cell count increases a little bit more than average viral load does, but differences are small and far from significant (confidence intervals overlap in figure 3).

The variance of the residual error term for CD4 T-cell count ranged from 0.91 before 1988 to 0.54 after 1991 (on the cube root scale). For viral load, the variance of the residual error term was 0.49 (on the base-10 logarithmic scale). Hence, using that 95% of the short term variation and measurement error is between the range -1.96 × standard deviation and +1.96 × standard deviation, on the logarithmic relative AIDS risk scale, this corresponds to 1.422 × 2 × 1.96 × 0.49 = 3.9 for viral load and 0.5801 × 2 × 1.96 × 0.54 = 1.7 to 0.5801 × 2 × 1.96 × 0.91 = 2.2 for CD4 T-cell count. Hence, CD4 T-cell count can be measured more reliably than viral load.

### Marker evolution before AIDS diagnosis

In the model of marker evolution during the four years preceding AIDS diagnosis, the fitted HIV RNA increase (on the base-10 logarithmic scale) was 0.167 (95% CI 0.101 to 0.231) per year from 4 to 1.5 years before AIDS diagnosis, and 0.223 (95% CI 0.138 to 0.314) per year for the last 1.5 years before AIDS diagnosis. The 0.057 change in slope between the two periods was not statistically significant (95% CI – 0.065 to 0.182). For CD4 T-cell count, on the other hand, the decline (on cube root scale) changed from -0.43 to -1.27 per year at 1.5 years before AIDS diagnosis, and this change in slope was significant (95% CI 0.66 to 1.02). When evaluated on the logarithmic relative AIDS risk scale, the RNA slope changes from 0.167 × 1.422 = 0.24 to 0.223 × 1.422 = 0.32, whereas the CD4 slope changes from 0.43 × 0.5801 = 0.25 to 1.27 × 0.5801 = 0.74. Hence, CD4 T-cell count and HIV RNA level show similar trends from 4 to 1.5 years before AIDS diagnosis, but during the last 1.5 years CD4 T-cell count changes more rapidly.

## Discussion

The aim of the study was to reconsider the existence of a viral load setpoint and compare it with CD4 T-cell evolution after HIV seroconversion. This was done by modeling average trajectories for both markers and presenting results on a common scale as determined by AIDS risk. The HIV RNA setpoint theory has been characterized by three aspects: levels after the initial peak are predictive for subsequent disease progression, a stable plateau phase and an increase shortly before AIDS diagnosis.

Higher predictive value of viral load shortly after seroconversion was not analysed here, since this has been shown convincingly before [11,12]. Note that also in the current analysis average HIV RNA level six months after seroconversion was more clearly associated with time to AIDS than CD4 T-cell count was. However, this result was based on a stratification of marker evolution by progression time to AIDS. In a genuine prediction model, stratification cannot be based on the future.

We modelled the longitudinal evolution of the markers using a scale that is related to AIDS progression. Our analysis showed that both markers revealed similar patterns over time after seroconversion: after a very gradual change in the first few years, the slope became increasingly steeper longer after seroconversion. The same similarity between both markers was found when patients were stratified by progression time. Persons with slower AIDS progression had more stable values for both markers during the first years. Although shapes were similar, the average decline in CD4 count corresponded to a much larger change in AIDS risk than the average increase in viral load did. This difference does not mean that CD4 count has the larger predictive value for AIDS progression. In our model, the markers were used as dependent variables and their evolution was modelled over time. Marker patterns were averaged over all individuals, also the ones who developed AIDS shortly after seroconversion. In a predictive model, a future AIDS event is the dependent variable, and prediction at some point after seroconversion is based on marker values for individuals who are still AIDS free. In the time-dependent Cox analysis (table 2), the markers were used as predictors, but this model was only used to determine an appropriate scale for the longitudinal marker analysis. Actually, except for the first two years after seroconversion, during which viral load had higher predictive value, the markers have been shown to have similar predictive value for the probability to develop AIDS within three years [12].

With respect to the third part of the setpoint theory, an increase in HIV RNA level was present already several years before AIDS diagnosis, and this slope did not change significantly at 1.5 years before AIDS diagnosis. On the other hand, a strong biphasic pattern was present for CD4 T-cell count.

For the modeling of the CD4 trajectories, the cube root transformation was chosen for its better fit with the random effects model we used. As the same scale turned out to be useful when modeling AIDS risk as a function of CD4 count, it was employed also to present results in figure 1. With different clinically relevant end points, like death risk or probability to develop AIDS within a certain time span, a different scale might be more appropriate to present results. However, any clinically relevant scale that represents AIDS or death risk will reflect that changes in CD4 count are more important at low counts than at high counts (see also Geskus et al. [12], in which a fourth root transformation was used).

We argue against the use of cross-sectional methods and scatterplots to describe the marker evolution in situations where marker values affect dropout rate. Results were strikingly different from the longitudinal approach. Since HIV infected individuals with low CD4 T-cell count or high viral load have a higher probability to die of AIDS, slow progressors are overrepresented later after infection, such that curves are biased upwards for CD4 T-cell count and biased downwards for viral load. Also, for studies on the effects of cofactors on marker evolution, the longitudinal random effects approach is preferred. For example, the cross-sectional approach may find no difference in CD4 values between two groups due to cancellation of effects: AIDS mortality at low CD4 count may be higher in the group with the steepest decline, such that the average CD4 value is equal in both groups. Although statistical analyses of marker patterns usually use some sort of longitudinal model, the cross-sectional approach has been applied as well. This holds not only for natural history studies, but also for studies of treatment effect in which part of the study group dies during the trial [13].

We modeled the simultaneous evolution of CD4 and viral load using follow-up data from seroconversion until AIDS diagnosis. The total follow-up is about equal to the median time to AIDS (18 individuals had more than ten years of follow-up). Hence, our results show marker evolution only during the first half of the time-to-AIDS distribution. However, the setpoint theory for viral load was based on data with similar follow-up and introduction of HAART has prevented study of longer-term evolution in a natural history setting.

## Conclusion

In summary, by using a common event (AIDS), we were able to directly compare evolution of CD4 T-cell count and HIV RNA level after HIV-1 seroconversion. Shortly after seroconversion, HIV RNA level is more predictive than CD4 T-cell count. However, a definition of setpoint based on the level reached shortly after the primary infection phase has little clinical relevance, since a date of seroconversion is unknown for most diagnosed HIV infected individuals. Also, the effect of sequential treatment interruptions cannot be evaluated based on the return to the personal setpoint level, if such level only exists shortly after seroconversion. Since both markers are frequently monitored as part of clinical care, more recent information on marker evolution is available. A setpoint, if defined as a stable level for several years, holds as much for CD4 T-cell count as for viral load, and only for a subgroup of HIV infected individuals. Such a setpoint does not preclude the need for frequent monitoring of viral load in making the decision to start HAART, since the stable phase may end at any time. Since CD4 T-cell count can be measured more reliably than viral load (a maximum of 2.2 versus 3.9), the end of the stable phase may be more dificult to detect for viral load, which makes frequent monitoring even more important for viral load than for CD4 T-cell count. The third aspect of the setpoint concerns the change from a stable phase to an increase in viral load shortly before AIDS diagnosis. We did not find a stable phase for any of the markers in the last four years before AIDS, but the evolution of CD4 T cell count is more biphasic than the evolution of HIV RNA.

## Methods

### Data

We used data from two different sources, the Amsterdam Cohort Study (ACS) among homosexual men and the French ANRS SEROCO Cohort Study. Informed consent was obtained from all participants.

Started in 1984, the ACS has required that participants be free of AIDS-defining conditions at entry. In our analysis, we included those with a period between the last HIV-seronegative test and the first HIV-seropositive test of not more than two years; we imputed their seroconversion date via conditional mean imputation [14]. Follow-up data from hospitals was added to ACS data.

The French SEROCO cohort started in 1988, has enrolled HIV-infected, non-haemophiliac adults referred from 17 hospitals and a network of private practitioners. For reasons of homogeneity, we analyzed only homosexual men from the cohort. Like the ACS men, they had no more than two years between the date of last HIV-seronegative test and date of first HIV-seropositive test. Their date of seroconversion had been imputed as described in Hubert et al. [15].

Data was drawn from the start of each study until HAART was widely introduced in the two countries: July 1st, 1996 in the Netherlands and February 1st, 1996 in France. In total, we used data from 400 persons (126 from Amsterdam and 274 from France). The same data were previously used to investigate the causal pathways of the effects of age and three genetic cofactors on AIDS development [16].

### Laboratory methods

All CD4 lymphocyte counts were obtained prospectively. In Amsterdam, they were measured in one laboratory, where single indirect immunofluorescence staining on Ficoll-isolated peripheral blood mononuclear cells was used until May 1988 and thereafter a double direct staining method. The Coulter Epics flow cytometer was used until 1991, then replaced by a FASCAN. Each day, CD4 samples were compared with values from healthy HIV-negative controls. In France, CD4 T-cell count measurements originated from 17 laboratories, so changes in method occurred more gradually. All HIV-1 RNA levels were determined retrospectively from stored sera. In France, the three participating university labs used reverse transcriptase-polymerase chain reaction (Amplicor HIV-1 Monitor assay, Roche Molecular Systems, Neuilly-sur-Seine, quantification threshold 200 copies/mL). In Amsterdam, one laboratory performed all HIV RNA assays. Most (83.6%) of the measurements were based on the NASBA technique (NASBA HIV-1 RNA QT; Organon Teknika, Boxtel, The Netherlands, quantification threshold 1000 copies/mL). The remaining were based on the Amplicor (2.4%) or the Nuclisens (14%) technique. Since the Amplicor test gives lower values, a correction factor was applied (on base-10 logarithmic HIV-RNA scale: 3.5–4.5: +0.04; 4.5–5.5: +0.22; >5.5: +0.29 [17]).

### Statistical methods

A bivariate quadratic random effects model was used to describe the simultaneous evolution of CD4 T-cell count and viral load after HIV-1 seroconversion (as in Geskus *et al*. [16]). The trajectory of each marker was thereby allowed to follow a polynomial trend over time since seroconversion, which can differ per individual. The six parameters per individual (the intercept, slope, and quadratic term for each marker) were assumed to originate from a multivariate normal distribution. The mean of this distribution yields the average trajectories at the population level. HIV RNA levels below the detection limit were treated as left-censored [18]. Since the viral load peaks shortly after infection, we allowed for a change in the slope of viral load evolution at six months after seroconversion (for CD4 T-cell count, no temporary drop was seen in our data). We only used data from the pre-HAART era. Still, less potent anti-retroviral therapy (ART) may have had some temporary effect on marker evolution. The effect of ART was incorporated as in Hubert *et al*. [7]. Zidovudine monotherapy was allowed to affect both marker levels for one year, with a change in effect after six months. In the case of dual therapy, the second period lasted for nine months instead of six. Contrary to Hubert *et al*., effect sizes were estimated from our data. We included a calendar time effect for both average CD4 T-cell count and the variance of the residual (measurement) error term. Changes in 1988 and 1991 for Amsterdam correspond with changes in laboratory methods. For France, where CD4 data originate from different laboratories, we assumed a change in 1991, resulting in about equal number of measurements in both periods. Moreover, we allowed each laboratory to measure, on average, a different value for CD4 T-cell count (random laboratory effect). Since HIV RNA values were measured retrospectively within a short time period in only a few laboratories, no change occurred in laboratory method. Therefore, we assumed the standard deviation for the residual error term of viral load to remain constant. However, we included a calendar period effect on the overall level, since viral load was measured retrospectively from stored samples that may yield different values depending on their age. To describe the simultaneous evolution of CD4 T-cell count and viral load in the four years preceding AIDS diagnosis, a linear random effects model was fitted to the marker data from the subset of individuals with an AIDS diagnosis. In order to detect a change in marker trend shortly before AIDS diagnosis, the slope was allowed to change at 1.5 years before AIDS diagnosis.

In all random effects models, marker values were transformed in order to provide a better fit: the base-10 logarithm of viral load and the cube root of CD4 T-cell count (CD4^1/3^) were used [19]. The clinically relevant marker scale on which results are evaluated was determined by modeling the effect of time-updated marker values on AIDS risk in a Cox proportional hazards model. Fitted marker values based on the random effects estimates were used, and functional form was established via low-rank thin-plate splines [20]. Marker evolution over time was depicted graphically such that changes in marker value that induce similar changes in relative AIDS risk have equal distance. Presence of a more or less stable level was investigated. Average curves were shown for the whole population and for four subgroups of individuals designated by their disease progression [9]: AIDS occurring <3.5 years, 3.5 to 7 years or >7 years after seroconversion, and AIDS-free for more than 9 years after seroconversion.

Also, results from this longitudinal approach, describing average marker *patterns*, were compared with our results from a repeated cross-sectional approach. The repeated cross-sectional approach summarizes the available marker *values *at each point in time for the individuals who are still alive and in follow-up: a least squares curve or a lowess curve is fitted through the scatterplot data [7,21,22]. In a discrete version of this approach, the mean or median marker value over time periods of equal length (usually a year) is computed and these values are connected over time [23-25]. Whereas the repeated cross-sectional approach quantifies the change in average marker value over time, the longitudinal approach quantifies the average change in marker value over time; note the different position of "average".

CD4 T-cell count and HIV RNA level cannot be compared directly. However, using our AIDS risk scale, the average trajectories of the two markers were compared in one graph.

We used a Bayesian approach for estimation of the parameters, starting with non-informative priors. We used a random effects selection model similar to the one used by Faucett & Thomas [26], except that ours included the evolution of viral load. Posterior distributions were obtained via Markov chain Monte Carlo methods, using the WinBUGS program [27,28]. Three chains were generated, based on different sets of starting values. Parameter estimates are the medians of the posterior distributions. The range from the 2.5% to the 97.5% quantile is used to quantify the uncertainty in the parameter estimates. This range can be interpreted as a 95% confidence intervals (CI) and will be referred to as such. If the value "zero" is outside this interval, the effect is seen as statistically significant.

#### Model in formula form

In formula form, the model for the concurrent evolution of the markers is

(CD4(t)1/3logRNA(t)10)=(a1i+b1i t+c1i t2+θ1cal+ζ1ART+ε1(t)a2i+b2i t+c2i t2+δ2 t I(t<0.5)+θ2cal+ζ2ART+ε2(t))

with

$$\begin{array}{c} {\left(a_{1}^{i},b_{1}^{i},c_{1}^{i},a_{2}^{i},b_{2}^{i},c_{2}^{i}\right)}^{T}\sim N({\left(\alpha_{1}(l),\beta_{1},\gamma_{1},\alpha_{2}^{\text{site}},\beta_{2},\gamma_{2}\right)}^{T},\Sigma )\\ \alpha_{1}(l_{F})\sim N(\mu_{F},\sigma_{\text{lab}}^{2})\\ \alpha_{1}(l_{A})=\mu_{A}\\ \varepsilon_{1}(t)\sim N(0,\sigma_{\text{cal}}^{2})\\ \varepsilon_{2}(t)\sim N(0,\tau^{2}) \end{array}$$

and all *ε*_1 _and *ε*_2 _independent. *ε*_1 _and *ε*_2 _model measurement error and short-term variation. The $a_{k}^{i}$, $b_{k}^{i}$ and $c_{k}^{i}$, *k *= 1, 2 describe the individual random effects. We allow the HIV-1 RNA level to have a different slope in the first six months after seroconversion (parameter *δ*_2_). Finally, the effects of calendar period and ART use on CD4 T-cell count and HIV-1 RNA level are represented by $\theta_{k}^{\text{cal}}$ and $\zeta_{k}^{\text{ART}}$.

The effect of the markers on the hazard of AIDS was described in a Cox model via

$$\lambda^{i}(t)=\lambda_{0}(t)\exp\{g(a_{1}^{i}+b_{1}^{i}t+c_{1}^{i}t^{2})+\text{h}(a_{2}^{i}+b_{2}^{i}t+c_{2}^{i}t^{2})\},$$

where *g *and *h *describe smooth trends on AIDS risk for CD4 T-cell count and HIV RNA level, using low-rank thin-plate splines [20].

## Competing interests

The author(s) declare that they have no competing interests.

## Authors' contributions

RG: main author, substantial contributions to conception and design, analysis and interpretation of data MP: substantial contributions to conception and design, analysis and interpretation of data JH: substantial contributions to analysis and interpretation of data FM: substantial contributions to conception and design and acquisition of data BB: substantial contributions to conception and design and acquisition of data CR: substantial contributions to conception and design JD: substantial contributions to conception and design LM: substantial contributions to conception and design, acquisition of data, and interpretation of data All authors read and approved the final manuscript.

## References

[B1] Henrard DR Phillips JF Muenz LR Blattner WA Wiesner D Eyster E Goedert JJ Natural History of HIV-1 Cell-Free Viremia JAMA 1995 274 554 558 10.1001/jama.274.7.554 7629984

[B2] Ho DD Viral Counts Count in HIV Infection Science 1998 272 1124 1125 10.1126/science.272.5265.1124 8638155

[B3] de Wolf F Spijkerman I Schellekens PT Langendam M Kuiken C Bakker M Roos M Coutinho R Miedema F Goudsmit J AIDS prognosis based on HIV-1 RNA, CD4+ T-cell count and function: markers with reciprocal predictive value over time since seroconversion AIDS 1997 11 1799 1806 10.1097/00002030-199715000-00003 9412697

[B4] Katzenstein TL Pedersen C Nielsen C Lundgren JD Jakobsen PH Gerstoft J Longitudinal serum HIV RNA quantification: Correlation to viral phenotype at seroconversion and clinical outcome AIDS 1996 10 167 173 8838704 10.1097/00002030-199602000-00006

[B5] Vidal C Garcia F Romeu J Ruiz L Miro JM Cruceta A Soriano A Pumarola T Clotet B Gatell JM Lack of evidence of a stable viral load set-point in early stage asymptomatic patients with chronic HIV-1 infection AIDS 1998 12 1285 1289 10.1097/00002030-199811000-00009 9708407

[B6] O'Brien TR Rosenberg PS Yellin F Goedert JJ Longitudinal HIV-1 RNA Levels in a Cohort of Homosexual Men J Acquir Immune Defic Syndr Hum Retrovirol 1998 18 2 155 161 9637580 10.1097/00042560-199806010-00007

[B7] Hubert JB Burgard M Dussaix E Tamalet C Deveau C Le Chenadec J Chaix ML Marchadier E Vildé JL Delfraissy JF Meyer L the SEROCO Study Group Natural history of serum HIV-1 RNA levels in 330 patients with a known date of seroconversion AIDS 2000 14 123 131 10.1097/00002030-200001280-00007 10708282

[B8] Sabin CA Devereux H Phillips AN Hill A Janossy G Lee CA Loveday C Course of Viral Load Throughout HIV-1 Infection J Acquir Immune Defic Syndr Hum Retrovirol 2000 23 2 172 177 10737432 10.1097/00126334-200002010-00009

[B9] Lyles RH Muñoz A Yamashita TE Bazmi H Detels R Rinaldo CR Margolick JB Phair JP Mellors John W for the Multicenter AIDS Cohort Study Natural History of Human Immunodeficiency Virus Type 1 Viremia after Seroconversion and Proximal to AIDS in a Large Cohort of Homosexual Men The Journal of Infectious Diseases 2000 181 872 880 10.1086/315339 10720507

[B10] Touloumi G Pantazis N Babiker AG Walker SA Katsarou O Karafoulidou A Hatzakis A Kholoud Porter on behalf of the CASCADE Collaboration Differences in HIV RNA levels before the initiation of antiretroviral therapy among 1864 individuals with known HIV-1 seroconversion dates AIDS 2004 18 1697 1705 10.1097/01.aids.0000131395.14339.f5 15280781

[B11] Mellors JW Muñoz A Giorgi JV Margolick JB Tassoni CJ Gupta P Kingsley LA Todd JA Saah AJ Detels R Phair JP Rinaldo CR Plasma Viral Load and CD4^+ ^Lymphocytes as Prognostic Markers of HIV-1 infection Annals of Internal Medicine 1997 126 946 954 9182471 10.7326/0003-4819-126-12-199706150-00003

[B12] Geskus RB Miedema FA Goudsmit J Reiss P Schuitemaker H Coutinho RA Prediction of Residual Time to AIDS and Death Based on Markers and Cofactors J Acquir Immune Defic Syndr Hum Retrovirol 2003 32 5 514 521 12679703 10.1097/00126334-200304150-00008

[B13] Morris L Martin DJ Bredell H Nyola SN Sacks L Pendle S Page-Shipp L Karp CL Sterling TR Quinn TC Chaisson RE Human Immunodeficiency Virus-1 RNA Levels and CD4 Lymphocyte Counts, during Treatment for Active Tuberculosis, in South African Patients Journal of Infectious Diseases 2003 187 1967 1971 10.1086/375346 12792875

[B14] Geskus RB On the inclusion of prevalent cases in HIV/AIDS natural history studies through a marker-based estimate of time since seroconversion Statistics in Medicine 2000 19 1753 1769 10.1002/1097-0258(20000715)19:13<1753::AID-SIM487>3.0.CO;2-F 10861776

[B15] Carré N Deveau C Belanger F Boufassa F Persoz A Jadand C Rouzioux C Delfraissy J Bucquet D Effect of age and exposure group on the onset of AIDS in heterosexual and homosexual HIV-infected patients AIDS 1994 8 6 797 802 8086139 10.1097/00002030-199406000-00012

[B16] Geskus RB Meyer L Hubert JB Schuitemaker H Berkhout B Rouzioux C Theodorou ID Delfraissy JF Prins M Coutinho RA Causal pathways of the effects of age and the *CCR5**-**32*, *CCR2-64I *and *SDF-1 3'A *alleles on AIDS development J Acquir Immune Defic Syndr Hum Retrovirol 2005 391 3 321 326 15980693 10.1097/01.qai.0000142017.25897.06

[B17] Annales du contrôle national de qualité Agence Française de securité sanitaire des produits de santé, No. 17 1999

[B18] Hughes JP Mixed effects Models with Censored Data with Application to HIV RNA Levels Biometrics 1999 55 625 629 10.1111/j.0006-341X.1999.00625.x 11318225

[B19] Taylor JMG Law N Does the Covariance Structure Matter in Longitudinal Modelling for the Prediction of Future CD4 Counts? Statistics in Medicine 1998 17 2381 2394 10.1002/(SICI)1097-0258(19981030)17:20<2381::AID-SIM926>3.0.CO;2-S 9819834

[B20] Crainiceanu C Ruppert D Wand M Bayesian Analysis for Penalized Spline Regression Using WinBUGS Journal of Statistical Software 2005 14 14 http://www.jstatsoft.org/

[B21] Margolick JB Donnenberg AD Muñoz A Park LP Bauer KD Giorgi JV Ferbas J Saah AJ the Multicenter AIDS Cohort Study Changes in T and Non-T Lymphocyte Subsets Following Seroconversion to HIV-1: Stable CD3^+ ^and Declining CD3^- ^Populations Suggest Regulatory Responses Linked to Loss of CD4 Lymphocytes Journal of Acquired Immune Deficiency Syndromes 1993 6 153 161 8094458

[B22] Margolick JB Muñoz A Donnenberg AD Park LP Galai N Giorgi JV O'Gorman MRG John Ferbas for the Multicenter AIDS Cohort Study Failure of T-cell homeostasis preceding AIDS in HIV-1 infection Nature in Medicine 1995 1(7) 674 680 10.1038/nm0795-674 7585150

[B23] Schellekens PTA Tersmette M Roos MTL Keet RP de Wolf F Coutinho RA Miedema F Biphasic rate of CD4^+ ^cell count decline during progression to AIDS correlates with HIV-1 phenotype AIDS 1992 6 665 669 10.1097/00002030-199207000-00008 1354447

[B24] Stein DS Korvick JA Vermund SH CD4+ lymphocyte cell enumeration for prediction of clinical course of human-immunodeficiency-virus disease – A review J Infect Dis 1992 165 352 363 1346152 10.1093/infdis/165.2.352

[B25] Multicohort Analysis Project Workshop Part II Bird G Cook R Deangelis D Farewell V Fielding K Fore A Gore S Kramer A Lee C McNeil A Pezzotti P Phillips A Raboud J Rezza G Sabin C Satten G Brettle R Hamilton B Povey S Raab G Richardson A Aiuti F Angarano G Barbanera M Canessa A Castelli F Gafa S Lazzarin A Muratori S Pristera R Ricchi E Salassa B Sinicco A Tirelli U Viale P Zaccarelli M Bofill M Elford J Janossy G Goedert J Yellin F Coates R Calzavarra L Reid S Immunological marker paths for seroconversion – single determinations of immunoglobulin-A and *β*_2_-microglobulin are not adequate to estimate time of HIV-infection AIDS 1994 8 923 933 7946101

[B26] Faucett CL Thomas DC Simultaneously Modelling Censored Survival Data and Repeatedly Measured Covariates: a Gibbs Sampling Approach Statistics in Medicine 1996 15 1663 1685 10.1002/(SICI)1097-0258(19960815)15:15<1663::AID-SIM294>3.0.CO;2-1 8858789

[B27] Spiegelhalter DJ Thomas A Best NG WinBUGS version 1.2 User Manual 1999 MRC Biostatistics Unit

[B28] Gilks WR Richardson S Spiegelhalter DJ Markov Chain Monte Carlo in Practice 1996 Chapman & Hall

